# The relationship between overprotective parenting, toothbrushing practices and children's behaviour during dental treatments in 4 to 11-year-old Dutch children: a cross sectional study

**DOI:** 10.1007/s40368-023-00814-1

**Published:** 2023-07-11

**Authors:** M. de Jong-Lenters, B. Pasman, D. Duijster

**Affiliations:** 1 Department of Paediatric Dentistry, Academic Center for Dentistry Amsterdam, University of Amsterdam and VU University, Gustav Mahlerlaan 3004, 1081LA Amsterdam, The Netherlands; 2 Department of Oral Public Health, Academic Center for Dentistry Amsterdam, University of Amsterdam and VU University, Gustav Mahlerlaan 3004, 1081LA Amsterdam, The Netherlands

**Keywords:** Overprotective parenting, Oral health behaviour, Dental health, Toothbrushing

## Abstract

**Purpose:**

Overprotective parenting and its impact on child development has gained increasing public attention. This study explored the association between overprotective parenting and behaviour during dental treatments and toothbrushing behaviour of 4 to 11-year-old-children.

**Methods:**

In this cross-sectional study, caregivers of 4-to-11-year-old children who visited a referral practice for dental treatment in Leiden, The Netherlands, completed a questionnaire about overprotective parenting, using the Parental Overprotection Measure (POM), and children’s toothbrushing behaviour. The dentist and dental assistant used the Venham scale to assess children’s behaviour during dental treatments. Associations between the POM and the Venham scale and toothbrushing variables, were analysed using multiple ordered logistic regression.

**Results:**

The sample included 96 children (mean age: 7.3 ± 2.1 years, 59 boys). Overprotective parenting (higher POM scores) was significantly associated with more disruptive behaviour of children during dental treatments (higher Venham categories) (OR: 1.08 (95% CI 1.04; 1.13)) and lower caregiver self-efficacy regarding toothbrushing (OR 0.96 (95% CI 0.93; 0.99)), after adjustment for confounders. No associations between overprotective parenting and toothbrushing frequency or skipping toothbrushing were found.

**Conclusions:**

Overprotective parenting has been associated with children’s negative behaviour during dental treatments and lower caregiver self-efficacy regarding toothbrushing in primary school children who are treated in a referral practice for paediatric dental care.

## Introduction

Since the new millennium, there has been growing interest in the potential impact of overprotective parenting on child development. Overprotective parents excessively regulate a child’s activities and routines, are highly controlling and create dependence on the parent(s) (Wood et al. 2003; Ungar 2009). This stems from the belief that the environment is full of danger and that children need to be spared from adversity, disappointment, and discomfort. Their intention is to keep children safe, not only physically but also emotionally. As a consequence, parents highly supervise their child and often do not entrust them with tasks and responsibilities (Ungar 2009) This hinders the child from developing resilience and learning how to deal with everyday challenges and anxiety-provoking situations (Spokas and Heimberg 2009; Gere et al. 2012).

Overprotection exists in every generation; however it has been more commonly reported in the past twenty years (LeMoyne and Buchanan 2011). Overprotective parenting has gained increasing public attention, inspiring a number of popular labels including ‘the curling parent’, ‘the lawnmower parent’ and ‘helicopter parenting’ (Hancock et al. 2014), since “obstacles are being cleared by the parent”. Also, scientific literature on overprotective parenting, often in relation to socio-emotional and behavioural development of children, has started to emerge. Studies have shown that overprotection by parents has been associated with internalising behaviour problems in children, including depression and anxiety, as well as externalising behaviour problems, such as attention deficit hyperactivity disorder and conduct disorder (Wood et al. 2003; Bayer et al. 2006; McLeod et al. 2007; Gere et al. 2012; Kiel and Maack 2012; Roo et al. 2022). In adolescence, lower levels of self-efficacy and somatic symptoms have been reported (Janssens et al. 2009; Giverts and Segrin 2014). In terms of health outcomes, Hancock et al. (2014) found that 10 to 11-years olds were more likely to develop obesity if the mother was overprotective.

Overprotective parenting may also indirectly affect children’s oral health. One hypothesized mechanism relates to disruptive behaviour during dental treatments. Uncooperative behaviour of the child, such as protesting, crying and body movement, could contribute to poor oral health, as it may lead to disruption or discontinuation of necessary dental treatment or preventive care (Hine et al. 2019). Children of overprotective parents may feel the concerns and fears of their parents when visiting the dentist, and copy these. Also, the protective behaviour of parents may have conditioned the child to express discomfort or protest in order to get out of uncomfortable situations, such as treatment at the dentist. Yet, no studies have been conducted to test these assumptions. Previous studies, however, have shown that solely the presence of parents in the treatment room can already negatively influence the child's cooperative behaviour while in the dental chair (Gerull and Rapee 2002; Cox et al. 2011). Therefore, it is common practice in many referral clinics for paediatric dental care that parents do not accompany their child during treatment.

A second mechanism, potentially linking overprotective parenting to children’s oral health, operates via children’s toothbrushing behaviour. Poorer oral hygiene behaviour, including lower toothbrushing frequency, was observed in families with poor family organisation (lack of structure and routines) and ineffective parenting, characterised by inconsistent discipline practices and low levels of positive involvement and reinforcement (Duijster et al. 2014; Kumar et al. 2017). Overprotective parenting in particular, has not yet been investigated in relation to children’s oral hygiene behaviour. However, it is plausible that overprotective parents are more likely to skip toothbrushing when their child is crying or heavily resisting. The aim of this study was to explore the association between overprotective parenting and behaviour during dental treatments of 4 to 11-year-old children in Leiden, The Netherlands. A second objective was to assess the association between overprotective parenting and children’s toothbrushing behaviour, including toothbrushing frequency, skipping toothbrushing and caregivers’ self-efficacy towards toothbrushing.

## Materials and methods

### Study design, sample and procedure

The cross-sectional study was conducted in 2021 and has been approved by the Ethical Committee of Academic Centre for Dentistry Amsterdam (ACTA) (protocol number 202076). Subjects were recruited from a referral practice for paediatric dental care in Leiden, The Netherlands. A referral practice was chosen for the purpose of this study because treatment needs of the patient population are generally higher and the provision of care is often more complex. As a result, more variation in children’s behaviour during dental treatments can be expected compared to general dental practice—including more extreme behaviour.

Children were eligible to participate if they were between 4 to 11 years old and were visiting the referral practice to undergo dental treatment, including restoration, extraction, endodontic treatment, sealants or a stainless-steel crown using the Hall method. As this was a first exploratory study to assess a possible association between overprotective parenting and children’ oral health variables, children of all primary school ages were included since caregivers play an influential role in children’s behaviours, including dental behaviours, during this age period. Children with a mental or physical disability were excluded. Caregivers of children had to be proficient in Dutch or English.

A researcher selected eligible patients from the dental schedule for that particular day. Dentists were informed by the researcher if they had a patient who was eligible for the study. When the child (patient) was called into the treatment room, the caregiver of the eligible child—who was staying in the waiting room—was informed about the study by the researcher. This was done intentionally in this order, so that the child would not be aware of the study, which could have affected his or her behaviour during treatment. After providing written consent, the parent was asked to complete a questionnaire in the waiting room. Meanwhile, the dentist and the assistant both observed and scored the behaviour of the child during dental treatments. If a caregiver did not provide consent, the forms with the behaviour scores were destroyed immediately afterwards. Since the aim of the study was exploratory, no a priori hypotheses were put forward on the size of the putative associations and therefore no power calculations were performed.

### Data collection

The primary dependent variable of this study was the child’s behaviour during dental treatments, measured with the Venham scale (Venham et al. 1980), through observation. Secondary dependent variables included toothbrushing frequency, skipping toothbrushing and the caregiver(s)’s self-efficacy with regard to brushing their children’s teeth, assessed via a self-reported questionnaire. The independent variable, overprotective parenting, was measured with the Parental Overprotection Measure (POM) questionnaire (Clarke et al. 2013).

#### Behaviour of the child during dental treatments

To measure the child’s behaviour during dental treatments, the Venham scale was used, which was developed in 1980 in the United States to observe and assess children’s responses to dental stress (Venham et al. 1980). The Venham scale has six categories: relaxed (‘0’), uneasy (‘1’), tense (‘2’), reluctant (‘3’), interference (‘4’) and out of contact (‘5’). The validity and reliability of the Venham scale has been demonstrated in the Dutch population (Aartman et al. 1998; Krikken 2013). The Venham scale was independently scored by a (paediatric) dentist and an assistant on a separate sheet. If the patient's behaviour changed during treatment or there was a short-term peak, a representative overall score was recorded after treatment was finished. To standardize scores as much as possible between multiple observers, an information meeting was organized for dentists and assistants prior to data collection.

#### Toothbrushing behaviour

Questions on toothbrushing behaviour were included in the questionnaire, using original questions developed by Pine et al. (2004) and Finlayson et al (2005). Toothbrushing frequency was measured using the question ‘How often do you brush your child's teeth?’ (response options: ‘never’, ‘not every day’, ‘once a day’, ‘twice a day’ and ‘three times a day’). These were subsequently categorized into ‘once or less per day’ and ‘twice or more per day’. Skipping tooth brushing was measured using the question ‘Is toothbrushing ever skipped? (response options: ‘always’, ‘usually’, ‘sometimes’ and ‘never’). The variable was subsequently dichotomized into ‘yes’ and ‘no’ (never). Caregivers’ self-efficacy with regard to brushing their children’s teeth was measured using a modified shortened questionnaire by Finlayson et al. (2005). The caregiver was asked how confident he or she would be in successfully brushing the child’s teeth, if ‘you are bothered by your crying child?’, ‘you are bothered because your child is not willing to cooperate with brushing?’ and ‘you are told by your child that he/she does not feel like brushing right now’. Response options for all three questions were ‘not at all certain’ (1), ‘not very certain’ (2), ‘reasonably certain (3), ‘very certain’ (4). The sum of the three questions was computed, with scores varying from 3 to 12, with higher scores indicating a higher self-efficacy. Self-efficacy was subsequently groups into four categories (‘very low’, ‘low’, ‘high’, ‘very high’) based on quartiles. Questions to measure the toothbrushing variables were translated into Dutch and back-translated and tested for validity in a Dutch sample of parents of 6-year-olds (Duijster et al. 2014).

#### Overprotective parenting

To measure the extent of overprotective parenting, the validated POM questionnaire was used (Edwards et al. 2010; Clarke et al. 2013). The questionnaire was translated into Dutch and then back-translated into English and sent to the first author of the team who developed the POM. The questionnaire includes 19 questions about overprotective parenting, which can be answered on a 5-point Likert scale ranging from 1 (‘not at all’) to 5 (‘very much/very often’). Example questions include: ‘I comfort my child immediately when he/she cries’, ‘I try to anticipate and avoid situations where my child might do something risky’, ‘I try to protect my child from making mistakes’ and ‘I will only leave my child with close friends or relatives if I have to go out’. Total scores can vary from 19 to 95, with a higher number indicating more overprotective parenting.

#### Sociodemographic covariates

Sociodemographic covariates included the child’s age and gender, the relationship of the caregiver to the child, education level of the caregiver, country of birth of the child, father and mother of the child, and birth order of the child. According to the parents’ countries of birth, children were categorized as ‘Dutch’, ‘migration background—European, and ‘migration background—non-European’. When one of the two parents was born in The Netherlands, the child was classified as Dutch. Parental education level was categorized into ‘low’ (‘no education’, ‘primary school’, ‘secondary school—lower level), ‘medium’ (‘secondary school—higher level’, ‘lower vocational education’) and ‘high’ (‘higher vocational education’, ‘university’).

### Statistical analysis

Data were analyzed using STATA (Release 16. College Station, TX: StataCorp LLC. StataCorp). POM scores were normally distributed. To assess the association between overprotective parenting and children’s behaviour during dental treatment, the mean POM scores were compared between the six Venham categories using the ANOVA test, and a Spearman correlation test was performed. Subsequently, ordered logistic regression with backward selection was performed to assess the association between POM scores and the Venham scale, adjusted for sociodemographic covariates. Only those covariates that were significantly associated with the Venham scale (p < 0.05) were retained in the model.

The associations between overprotective parenting and toothbrushing frequency and skipping toothbrushing were analysed by comparing the mean POM scores between groups using the independent samples T-test. The association between POM scores and parental self-efficacy was tested using the Spearman correlation test. The latter (significant) association was subsequently adjusted for demographic covariates using ordered logistic regression with backward selection.

## Results

Caregivers of 98 eligible children were invited to participate, of which two declined (response rate = 98.0%). The characteristics of the study sample are shown in Table 1. A total of 96 children (59 boys, 61.5%) aged between 4 and 11 years (mean 7.3 ± 2.1 years) took part in this study. Half the caregivers were highly educated (52.2%) and the majority of children had caregivers of Dutch origin (69.5%). The distribution of the Venham categories and the toothbrushing variables are shown in Table 2. The mean POM score was 51.2 ± 12.9 (range: 19, 87).

**Table 1:** Sociodemographic characteristics of the study sample

	Mean ± sd (minimum, maximum)
Age (years)	7.3 ± 2.1 (4, 11)
	**n (%)**
Gender	
Girl	37 (38.5)
Boy	59 (61.5)
Parental level of education	
Low	18 (20.0)
Average	25 (27.8)
High	47 (52.2)
Ethnicity	
Dutch	66 (69.5)
Migration background—European	5 (5.3)
Migration background—Non-European	24 (25.3)
Birth order	
1st born	40 (42.1)
2nd born	42 (44.2)
3rd born	8 (8.4)
4th born or over	5 (5.3)

**Table 2:** Mean POM scores by Venham scale and toothbrushing variables

	Overprotective parenting (POM scores)	
	Mean ± sd (min, max)	p
Children’s behaviour during dental treatments (Venham scale)		< 0.001^a^
0 (n = 43)	46.7 ± 11.2	
1 (n = 30)	50.0 ± 10.9	
2 (n = 8)	57.4 ± 10.9	
3 (n = 4)	47.0 ± 6.2	
4 (n = 5)	60.4 ± 11.6	
5 (n = 6)	75.5 ± 9.6	
Toothbrushing frequency		0.145^b^
Once a day or less (n = 24)	54.5 ± 13.6	
Twice a day or more (n = 72)	50.0 ± 12.6	
Skipping toothbrushing		0.672^b^
Yes (n = 51)	50.6 ± 12.8	
No (n = 45)	51.8 ± 13.1	
Self-efficacy regarding toothbrushing		< 0.001^a^
Very low (n = 16)	49.5 ± 14.6	
Low (n = 27)	59.5 ± 15.0	
High (n = 28)	48.4 ± 8.4	
Very high (n = 23)	46.0 ± 10.1	

^a^ANOVA Test, ^b^Independent Samples T-test

In total, 17 extractions, 33 non-invasive treatments (sealants or Hall crowns) and 46 restorative invasive treatments (restorations) were performed. The type of treatment was not significantly associated with the Venham scale; mean Venham scores were 1.2 ± 1.7, 0.9 ± 1.5 and 1.2 ± 1.4 for extractions, non-invasive treatments and restorative-invasive treatments, respectively (p = 0.675). Hence, the type of treatment was not included in further analyses. The inter-rater reliability of the Venham scale of the dentist and assistant was high; in 97 out of the 98 participants, both dentist and the assistant filled in the same Venham category (intraclass correlation coefficient = 0.99). In the single case of disagreement, the score of the dentist was used for the analysis.

### Overprotective parenting and children’s behaviour during dental treatments

Overprotective parenting was significantly associated with more disruptive behaviour of children during dental treatments. POM scores were significantly lowest in children with a Venham category of ‘0’ (46.7 ± 11.2), and they were highest in those with a Venham category of ‘5’ (75.5 ± 9.6) (Table 2). The Spearman correlation for the association between POM scores and the Venham scale was r = 0.41 (p < 0.001). When looking at the separate age groups, the association was also significant for 4 to 5-year-old children (r = 0.51, p = 0.021, n = 20), 8 to 9-year-old children (r = 0.63, p = 0.001, n = 23) and 10 to 11-year-old children (r = 0.42, p = 0.082, n = 18), but not for 6 to 7-year-old children (r = 0.14, p = 0.437, n = 35).

In the ordered logistic regression model, a higher POM score was significantly associated with 1.08 (95% CI 1.05; 1.12) higher odds of having a higher Venham category (Table 3). This association did not change after adjustment for sociodemographic confounders. In the fully adjusted model, the odds of having a higher Venham category were significantly lower for children with medium educated parents, compared to those with low educated parents. Also, being further in line in birth order was associated with higher odds of having a higher Venham category.

**Table 3:** Ordered logistic regression for the association between POM scores and Venham scale, adjusted for sociodemographic covariates

	Children’s behaviour during dental treatments (Venham scale)			
	Crude model		Adjusted model	
	OR (95% CI)	p	OR (95% CI)	p
Overprotective parenting (POM scores)	1.08 (1.05; 1.12)	< 0.001	1.08 (1.04; 1.13)	< 0.001
Age (years)	0.88 (0.74; 1.06)	0.176		
Gender				
Girl	ref			
Boy	1.96 (0.90; 4.24)	0.088		
Parental education level				
Low	ref		ref	
Medium	0.16 (0.05; 0.52)	0.002	0.21 (0.06; 0.73)	0.014
High	0.19 (0.07; 0.54)	0.002	0.45 (0.14; 1.41)	0.170
Ethnicity				
Dutch	ref			
Migration background—European	0.52 (0.09; 3.01)	0.468		
Migration background—Non-European	2.81 (1.15; 6.87)	0.024		
Birth order				
1st born	ref		ref	
2nd born	3.20 (1.35; 7.58)	0.008	3.61 (1.40; 9.28)	0.008
3rd born	7.22 (1.94; 26.78)	0.003	3.78 (0.74; 19.33)	0.110
4th born or over	36.81 (4.06; 333.50)	0.001	22.18 (2.76; 178.59)	0.004

Ordered logistic regression

### Overprotective parenting and toothbrushing behaviour

Overprotective parenting was not associated with children’s toothbrushing frequency and skipping toothbrushing. Mean POM scores did not significantly differ between the toothbrushing groups (Table 2). However, higher POM scores were significantly associated with a lower self-efficacy regarding toothbrushing (Spearman correlation r = − 0.23, p = 0.026). This was also significant for 4 to 5-year-old children (r = − 0.46, p = 0.048) and 10 to 11-year-old children (r = − 0.50, p = 0.050). In the ordered logistic regression model, a higher POM score was significantly associated with 0.97 (95% CI 0.94; 0.99) lower odds of having a higher self-efficacy with regard to toothbrushing. This association did not alter after adjustment for significant covariates (OR: 0.96, 95% CI 0.93; 0.99). In the full model, parents with a medium or high education level had significantly higher odds of having a higher self-efficacy with regard to toothbrushing, compared to parents with a low education level.

## Discussion

This study found an association between overprotective parenting and behaviour during dental treatments of primary school children in The Netherlands; children with a more overprotective upbringing showed more uncooperative behaviour during dental treatment. Caregivers with an overprotective parenting style also had a lower self-efficacy with regard to toothbrushing when their child is uncooperative, crying or saying that he or she does not want to brush. No associations between overprotective parenting and the frequency of toothbrushing or skipping toothbrushing were found.

To the knowledge of the authors, this is the first study that explored overprotective parenting in relation to children’s oral health behaviours. Yet, information on this relationship could be obtained—to some extent—from existing dental literature on parenting. A few studies have shown that ineffective parenting, in terms of low warmth and involvement and inadequate discipline practices, was associated with poorer toothbrushing behaviours in children. Duijster et al. (2014) showed that children of families that lack structure and routines and have unclear roles and boundaries have lower toothbrushing frequency and are less likely to be supervised during toothbrushing by the parent. In addition, Abegg et al. (2000) demonstrated that a certain extent of flexibility is important to ensure regular tooth brushing. According to Kumar et al. (2017), coercive parenting practices (high levels of control) have a negative association with children’s toothbrushing behaviour. In a broader health context, there is a rich body of research that has demonstrated the influence of ineffective parenting practices on children’s general health, including externalising problem behaviour, childhood obesity and an unhealthy diet, such as lower fruit and vegetable consumption, higher caloric intake and lower frequency of eating breakfast (Ventura and Birch 2008; Shaffer et al. 2013).

Parenting styles are generally studied using Baumrind’s typology of authoritative, permissive, and authoritarian parenting. A number of studies have explored Baumrind’s parenting styles in relation to children’s behaviour during dental treatment. Aminabadi and Farahani (2008) showed that children of authoritarian and permissive caregivers showed significantly higher levels of discomfort during restorative dental treatment, compared to children of authoritative caregivers. Also Howenstein et al. (2015) showed that children of authoritative parents exhibited more positive behaviour in the dental chair than those of authoritarian and permissive parents. However, studies by Krikken et al. (2008) and Aminabadi et al. (2012) found no association between Baumrind’s parenting styles and dental behaviour management problems in children. To place overprotective parenting in the framework of Baumrind’s parenting styles is challenging, because overprotection combines aspects from different parenting styles. Overprotective parenting has overlap with authoritarian parenting (characterized by as a style that is restrictive, overly demanding, but lacking warmth and communication), and permissive parenting (defined as a style with high responsiveness to children’s needs, but with low parental self-efficacy and low boundary setting).

There are a number of strengths and limitations of this study that should be considered in the interpretation of findings. Strengths of this study were the high response rate and the use of validated instruments to measure the dependent and independent variables. Only two caregivers did not agree to participate in this study, suggesting little risk of selection bias. The Venham scale to measure children’s behaviour during dental treatments, has been shown to be a valid and reliable instrument which can be easily integrated into clinical practice (Moura et al. 2016). Observation was done by both the dentist and the assistant independently and the inter-rater reliability was high. The POM questionnaire is also a valid and psychometrically sound instrument to measure overprotective parenting (Edwards et al. 2010). The wide variety of scores in this study (ranging from 19 to 87 out of a possible range from 19 to 95) indicates that the POM is able to discriminate between different levels of overprotective parenting.

Yet, the study also had a number of limitations. Firstly, caregivers’ self-report could be biased by their own beliefs and perspectives, and there is a tendency for caregivers to answer questions in a socially desirable manner. The same holds for the measurement of toothbrushing variables using questionnaires. Caregivers of older children in the sample might not brush the teeth of their children anymore (despite the recommendation in Dutch guidelines to brush or re-brush by a parent up to the age of 10 years), and therefore it may be less relevant that caregivers answered questions on toothbrushing. Caregivers who have been registered at the referral practice for a longer period would have been informed about adequate oral hygiene measures and might have therefore overreported desirable toothbrushing behaviour.

Secondly, children in the study sample were recruited from a referral practice for paediatric dental care. It is likely that children in referral practices show more variation in behaviour during dental treatment (including more extreme uncooperative behaviour) due to higher levels of anxiety and complex oral treatment need in this patient population. This made the study sample suitable for the purposes of this study. However, the sample is not representative of the Dutch population, since most children are treated in general dental practice. Therefore, the external validity of the study is limited. Also, (paediatric) dental professionals working in a referral practice generally have more behavioural management skills, which could have influenced the child’s behaviour during dental treatment. Children are more likely to show discomfort during their first dental visit(s). The level of discomfort reduces when they are familiarised with a dental setting (after stepwise exposure), and trust has been built between the child and the dental professional (American Academy of Pediatric Dentistry 2011). Therefore, more cooperative behaviour can be expected after a child has had multiple sessions with the same dentist. The number of visits the child has had with the dentist—at the time of the study—was however not taken into account, and thus could have introduced bias (Table 4).

**Table 4:** Ordered logistic regression for the association between POM scores and parental self-efficacy regarding toothbrushing, adjusted for sociodemographic covariates

	Parental self-efficacy regarding toothbrushing (4 quartiles: very low, low, high, very high)			
	Crude model		Adjusted model	
	OR (95% CI)	p	B (95% CI)	p
Overprotective parenting (POM scores)	0.97 (0.94; 0.99)	0.016	0.96 (0.93; 0.99)	0.031
Age (years)	1.05 (0.88; 1.26)	0.591		
Gender				
Girl	Ref			
Boy	0.76 (0.36; 1.60)	0.473		
Parental education level				
Low	Ref		Ref	
Medium	4.55 (1.47; 14.09)	0.009	3.64 (1.16; 11.41)	0.027
High	2.93 (1.05; 8.17)	0.040	2.23 (0.78; 6.36)	0.132
Ethnicity				
Dutch	Ref			
Migration background—European	1.47 (0.33; 6.55)	0.608		
Migration background—Non-European	0.38 (0.15; 0.97)	0.043		
Birth order				
1st born	Ref			
2nd born	0.69 (0.31; 1.51)	0.354		
3rd born	0.08 (0.02; 0.38)	0.002		
4th born or over	0.14 (0.02; 1.07)	0.058		

Ordered logistic regression

Thirdly, a limitation was the small sample size and wide age range of children included in the study. Children’s cognitive, socioemotional, and behavioural development varies substantially between the ages of 4 to 11 years, which could have an importance influence on the association between overprotective parenting and children’s behaviour during dental treatment and toothbrushing behaviour. Since this was an exploratory study, the full age-range of primary school children was included. Analysis of sub age groups in this study suggests that overprotective parenting could be associated with children’s dental behaviour at both young ages (4–5 years) and older ages (10–11 years), however more research using larger sample sizes with a narrower age range are necessary to draw further conclusions on this.

Lastly, the study’s cross-sectional design has its limitations. No evidence can be provided on causal relationships or temporal precedence of variables. Parenting is a dynamic process that is subject to continuous change. Transitions that occur in families, such as the ageing of children, changes in family member composition, and life events, require caregivers to adapt to maintain ongoing functions. Therefore, parenting at the time of measurement of the study may not have been a representative reflection of parenting behaviours over the past and coming years. This would argue for future research with a longitudinal design.

## Conclusion

Considering any limitations of the present study the following conclusions can be made:

- overprotective parenting was associated with more disruptive behaviour during dental treatments of primary school children who are being treated in a referral practice for paediatric dental care in The Netherlands.
- Overprotective caregivers had lower self-efficacy with regard to brushing their children’s teeth.
- No significant differences were found between overprotective parenting and the frequency of toothbrushing and skipping toothbrushing.
- Further research is needed to determine to what extent the same associations is found in the general population and in which specific age groups.
- Results may indicate that less parental involvement during children’s dental treatment, for example by asking parents to remain in the waiting room, could benefit the dental procedure, especially in children from a high risk population.

## Data Availability

The data are available from the corresponding author upon reasonable request.
